# Usage trends for memory and vitality-enhancing medicines: A pharmacoepidemiological study involving pharmacists of the Gujarat region

**DOI:** 10.4103/0974-7788.72484

**Published:** 2010

**Authors:** Jigna Samir Shah, R. K. Goyal

**Affiliations:** *Department of Pharmacology, Shri Sarvajanik Pharmacy College, Mehsana - 384 001, India*; 1 *Vice-Chancellor, The Maharaja Sayajirao University of Baroda, Vadodara, Gujarat, India*

**Keywords:** Memory-enhancing medicines, polyherbal formulations, use of herbal medicines, vitality-enhancing medicines

## Abstract

**Objective:**

The aim of the study was to explore the trends and rationale of use of memory and vitality-enhancing medicines (MVEM) in the Gujarat region.

**Materials and Methods:**

A prospective pharmacoepidemiological study involving pharmacists of Gujarat region was carried out in the year 2005. Pharmacists (n = 351) working in general and Ayurvedic medical stores were selected from 12 districts of Gujarat region. The pharmacists were explained about the objective of the study and were given a pretested, validated questionnaire.

**Outcome Measures:**

The questionnaire included the questions regarding herbal MVEM used most commonly, percentage sale of herbal MVEM – sold with or without prescriptions – age group of patients and professional groups who used these drugs most commonly.

**Results:**

The number of individuals using MVEM was highest in the age group of 11–20 years (17.54%), followed by the 21–40 years group (17.12%), supporting the results that the professional group of students (17.29%) and the persons of business or service class (15.29%) are the highest users of these medicines. Evaluation of various constituents in the marketed polyherbal MVEM revealed that Brahmi (*Bacopa monniera*), Shankhpushpi (*Evolvulus alsinoides*), Ashwangandha (*Withania somnifera*), Jatamansi (*Nardostychos jatamansi*), Vacha (*Acorus calamus*) and Amla (*Phyllanthus emblica*) were the common ingredients in the polyherbal preparations.

**Conclusions:**

This study highlights commonly used Ayurvedic medicines that can be explored for safely enhancing memory and vitality performance. Hence, detailed and scientifically designed research on these drugs would help to identify safe and effective drugs for enhancing the same.

## INTRODUCTION

An enhanced life expectancy in developed countries has been accompanied by an increased number of people suffering from age-associated dementia. This can impair quality of life due to associated health problems, which also places a burden on patients and the state. Epidemiological studies in the Indian population reveal that dementia is largely a hidden problem in the country. Prevalence rates for dementia increase essentially with advancing age.[1] Persons above 60 years of age show 0.43% prevalence whereas persons aged above 65 years show 2.44% prevalence. The prevalence rate rises to 54.8% in individuals above 95 years of age.[2]

Herbal medications are sought by this group of patients in view of the lack of safe and effective medicines from conventional medicine.[3] Increasingly, persons who have no diagnosed medical or mental health condition are utilizing prescription drugs that were originally developed to improve executive function or memory in persons diagnosed with disorders such as attention deficit hyperactivity disorder or Alzheimer’s disease.[4–6] Evidence suggests that this practice, now known as neuroenhancement or cognition enhancement, is gathering momentum.[7,8] Evidence also suggests that these medications can improve memory and executive function in normal individuals.[9–11] However, other evidence suggests that these effects are complex, may not be uniformly positive across all dose levels or age groups and do not enhance all aspects of executive function or memory.[12,13]

Several studies have found that multi-vitamin/multi-mineral supplements can improve immunity in older people and several measures of wellbeing in adult healthy men.[14–20] General nutritional supplements may also help improve response to stress.[21] “Vitality enhancement” is applied to the use of interventions for normal ageing and in normal people for nonmedical purposes. In the last decade, healthy adult persons are increasingly using prescription drugs for memory and vitality enhancement.[22]

Ayurveda describes ‘*Rasayana chikitsa*’ (rejuvenation therapy[23] in which Rasayana drugs are used to modulate the neuro-endocrino-immune systems and have been found to be a rich source of antioxidants.[24,25] Ayurveda claims that several plants, the “Medhya” plants (intellect promoting), and herbs such as *Convolvulus microphyllus (C. pluricaulis), Centella asiatica, Bacopa monnieri, Acorus calamus, Zingiber officinale* and *Celastrus paniculatus* are beneficial in memory disorders.[26] The term “memory enhancement” is used for the use of various strategies to boost cognitive functions.[21]

Preliminary pharmacoepidemiological studies revealed that herbal memory and vitality-enhancing medicines (MVEM) are becoming very popular among the Indian population. The herbal drugs commonly used include Amalaki (*Emblica officinalis*), Brahmi (*Bacopa monniera*), Punarnava (*Boerhaavia diffusa*), Mandukaparni (*Hydrocotyle asiatica*), Ashwagandha (*Withania somnifera*), Galo (*Tinospora cordifolia*), Yashti-madhu (*Glycyrrhiza glabra*), Shankhapushpi (*Convolvulus pluricaulis and Evolvulus alsinoids*), Vacha (*Acorus calamus*) and Shatavari (*Asparagus racemosus*).[27]

The growing popularity of drugs that stimulate cognitive function, widespread public enthusiasm for products that seek to produce this effect, the growth of this segment in the pharmaceutical industry, absence of data on use of these medicines and the incomplete nature of information on the long-term side-effects on the nervous system prompted us to carry out a pharmacoepidemiological study among pharmacists to find out the usage trends and rationale for herbal MVEM being used more commonly in the Gujarat region.

## MATERIALS AND METHODS

### Design

A prospective, pharmacoepidemiological study involving pharmacists of Gujarat region was carried out in the year 2005.

### Setting/Location

The pharmacists working in pharmacy stores from 12 districts of Gujarat region (Ahmedabad, Mehsana, Vadodara, Patan, Surat, Banaskantha, Gandhinagar, Anand, Rajkot, Jamnagar, Bhuj and Junagadh) were selected. Both general medical and Ayurvedic medical stores were included from the rural as well as urban regions in the study.

### Subjects

Three hundred fifty-one (351) pharmacists were selected based on the fact that they worked in a store manned by a qualified pharmacist or qualified Ayurvedic personnel on a full-time basis. The stores run by other personnel like relatives of the pharmacist or part-time pharmacists were excluded from the study.

### Methods

The pharmacists at the medical stores were explained the objective of the study and were given a pretested, validated questionnaire.

### Outcome Measures

The questionnaire included questions like which herbal MVEM were used most commonly, what was the percentage sale of herbal MVEM, what was the percentage of sale of allopathic MVEM, whether they were sold through prescriptions or without prescriptions, which age group used these drugs most commonly, which professional group used MVEM most commonly, what were the instructions normally given to the patients by the pharmacists and which were the commonly prescribed drugs and brands in herbal MVEM.

They were also asked to get the information like whether the person buying the drug was using some other allopathic/herbal medicines also, whether he/she was really suffering from some ailment or whether they were consuming these medicines just for the sake of wellbeing or because someone like their friend or relative’s experience or some advertisement had encouraged them to use these medicines. This information was collected in the follow-up interview after 1 month.

## RESULTS

Of the 351 pharmacists, 284 pharmacists interviewed were from general medical stores while 67 personnel were from Ayurvedic stores. It was found that most of the polyherbal formulations of cognition enhancers were sold without valid physicians’ prescription (65%) in the rural area and 46% in the urban area of Gujarat region from the Ayurvedic or general medical stores. Interestingly, 65% of the pharmacists provided counseling to all their customers regarding herbal products. This included instructions like take the medicine with milk at night, do not take the medicine on empty stomach, etc.

The proportion of persons using MVEM was highest in the age group of 11–20 years (17.54%), followed by the 21–40 years group (17.12%), supporting the results that the professional group of students (17.29%) and the persons of business or service class (15.29%) are the highest users of these medicines [Table 1].

**Table 1:** Epidemiological surveys of age group and professional group

Parameter	Rural	Urban
% of persons in age group		
11–20	19.41	14.83
21–40	16.5	18.58
41–60	11.08	12
60 up	4.5	4.75
% of persons in professional group		
School/college	14.83	19.75
Housewives	5.08	5.16
Business/service	13.5	17.08
Geriatric	6.58	8.16

From a total of 45 herbal drugs [Table 2] that were found to be used commonly as memory and vitality enhancers, 25 cognition-enhancing polyherbal formulations [Table 3] were studied in some detail. Evaluation of various constituents in the marketed herbal MVEM revealed that Brahmi (*Bacopa monniera*), Shankhpushpi (*Evolvulus alsinoides*), Ashwangandha (*Withania somnifera*), Jatamansi (*Nardostychos jatamansi*), Vacha (*Acorus calamus*) and Amla (Embelica officinalis) were commonly used herbs in several herbal preparations [Table 4]. In Gujarat region, the leading brands in the market survey were found to be Mentat (The Himalaya Drug Company), Shankhpushpi (Zandu) and Shankhpushpi syrup (Unjha Pharmacy), etc. Although many herbal vitality-enhancing formulations are available in the market, 12 herbal formulations [Table 5] were found to be used most commonly as vitality-enhancing medicines.

**Table 2:** Herbal drugs commonly used to increase memory and vitality

Botanical name	Common name	Botanical name	Common name
*Achyranthes aspera*	*Chirchita*	*Narcissus pseudonarcissus*	*Daffodil*
*Acorus calamus*	*Vacha*	*Nardostachys jatamansi*	*Jatamansi*
*Artemisia nilagirica*	*Titapati*	*Nicotiana tobaccum*	*Tobacco*
*Artemisia vulgaris*	*Magdana*	*Ocimum basilicum*	*Kali Tulsi*
*Albizzia lebbeck*	*lebbeck Siris*	*Paeonia lactiflora*	*Paeony Root*
*Allium sativum*	*Lehsun*	*Paenia suffruticosa*	*Paeony*
*Angelica sinensis*	*Dong Quai*	*Panax ginseng*	*Ginseng*
*Bacopa monnieri*	*Brahmi*	*Pladera sessiliflora*	*Shankh-pushpi*
*Celastrus paniculatus*	*MaalKangni*	*Punica granatum*	*Pomegranate*
*Centella asiatica*	*Brahmi-munduki*	*Rhodiola rosea*	*Rose root*
*Chlorophytum arundinaceum*	*Safed musli*	*Rhodiola sachalinensis*	*Rhodiola root*
*Commiphora mukul*	*Guggal*	*Ricinus communis*	*Castor*
*Clitoria ternatea*	*Aprajita*	*Rosmarim officinalis*	*Rosemary*
*Convolvulus pluricaulis*	*Shankhpushpi*	*Rauwlfia serpentina*	*Sarpagandha*
*Cordydalis flexuosa*	*China blue*	*Salvia officinalis*	*Sage*
*Elettaria cardamom*	*Elaichi*	*Strychnos nux-vomica*	*Nux vomica*
*Elutherococcus senticosis*	*Siberian Ginseng*	*Terminalia belirica*	*Bibhitaki*
*Huperzia serrate*	*Qing Ceng Ta*	*Tinospora cordifolia*	*Guduchi*
*Hypericum perforatum*	*St. John’s wort*	*Uncaria tomentosa*	*Cat’s claw*
*Indigofera tinctoria*	*Nil*	*Valeriana wallichi*	*Tagara*
*Lawsonia inermis*	*Henna*	*Withania somnifera*	*Ashwagandha*
*Moringa oleifera*	*Ben tree*	*Zingiber officinale*	*Ginger*
*Melissa officinalis*	*Lemon balm*	**	**

**Table 3:** List of Ayurvedic formulations commonly available in the market and used to enhance memory

Formulation	Company
*Shankhpushpi*	Zandu
*Shankhpushpi*	Baidyanath
*Shankhpushpi syrup*	Unza Pharm
*Tejras*	Sandu
*Braintab*	Baidyanath
*Brento*	Zandu
*Mentat tablets/syrup*	Himalaya
*Smaran capsules*	Jamna
*Memorin capsules*	Phyto
*Brahmivita granules*	Bajaj
*Shankhpushpi*	Sakar
*Saraswstarishta*	Dabur
*Remem syrup/capsules*	Zydus Cadila
*Brahmibati*	Baidyanath
*Alert syrup*	Vasu Pharm
*Promind*	Lumen Marketing
*Brahmi Churna*	Bhuvaneshvari
*SwarnaBrahmivati*	Bhuvaneshvari
*Braintone tablets*	Multani Pharma
*Memorance*	Stallion Lab.
*Smrutil tablets*	Harinarayan
*Saraswatarishta*	Baidyanath
*Shankhpushpi*	Dabur
*Manoll syrup/capsules*	Charak
*Shantisagar*	Ratan Pharm

**Table 4:** List of herbs widely used as memory enhancer in herbal formulations

Plants	Number of formulations containing the herb/total number of formulations
*Evolvulus alsinoides*	20/25
*Bacopa moniera*	20/25
*Withania somnifera*	5/25
*Acorus calamus*	5/25
*Asparagus racemosus*	3/25
*Nardostachos jatamansi*	3/25
*Glycyrrhiza glabra*	3/25

**Table 5:** List of Ayurvedic formulations available in the market and used to enhance vitality

Formulation	Company
*Chyawanprash*	Baidyanath
*Sonachandi Chyawanprash*	Dabur India
*Zandu Chyavanprash*	Zandu
*Chyavanprash special*	Sandu
*Ashwagandha pills/ghrita/Arishta/churna*	Baidyanath
*Manmath ras*	Baidyanath
*Alpitone*	Zandu
*Shaktivita*	Painol Herbal and Co.
*Kesarijivan*	Zandu
*Smarton*	Indian Med. Pharm.
*Vitalfit*	Wings Pharma.
*Rasayanprash*	Chakrapani Ayurved

## DISCUSSION

Seventy-five percent to 80% of the world population depends on crude plant drug preparations or labeled herbal drugs to treat their health problems because of economic considerations. The present global market for these products has been estimated to be approximately 20 billion US dollars and is growing at the rate of 15–20% annually. Thus, plant-based therapeutic agents continue to have scientific, social and commercial significance and appear to be gathering a momentum in health-relevant areas. Hence, detailed and scientifically designed research on these plants would help to identify safe and effective drugs for memory enhancement.[28]

Analysis of the results of our study revealed that maximum people using MVEM were between the ages of 11 and 20 years (17.54%), followed by the ages of 21 and 40 years (17.12%). Results also showed that students (17.29%) and the persons of business or service class (15.29%) were the highest users of these medicines.

This is an age in which the values of performance, efficiency, improvement and self-realization are ever-present. Good physical and mental health is therefore considered a major asset in society.[29] With the explosion of information and advancement of industrialization, the students as well as the persons of business or service class are resorting to MVEM to improve and enhance attention, memory, vitality and other measures of wellbeing. The patients opt for herbal MVEM considering that they have fewer side-effects.[26] Also, use of MVEM by their friends or relatives might have prompted them to prefer the same.

Although the pharmacists normally do not provide counseling to their customers, we observed that 65% pharmacists showed interest in providing counseling to all their customers if suitable information is given by the manufacturers. It was found that most of the polyherbal formulations of cognition enhancers were sold without valid physicians’ prescription (65%) in the rural area and 46% in the urban area of Gujarat region from the Ayurvedic or general medical stores. Majority of people who use herbal medicines or over-the-counter medicines do not reveal to their physician or pharmacists and may have the side-effects from the interactions between herbal components and concurrent pharmacotherapy.[30] Therefore, it is important for the pharmacist to take up the responsibility of patient counseling and patient awareness regarding the drug-drug interactions strongly, especially if the drug is sold without prescription. This work does not address “nonmedical use of prescription nootropics/stimulants,”[31] a subject that may require a separate research.

We found that 45 herbal drugs were used alone or in combination in the form of extract, tea or powder as MVEM in Gujarat region. Evaluation of various constituents in the 25 marketed herbal cognition enhancers revealed that Brahmi (*Bacopa monniera*), Shankhpushpi (*Evolvulus alsinoides*), Ashwangandha (*Withania somnifera*), Jatamansi (*Nardostychos jatamansi*), Vacha (*Acorus calamus*) and Amla (*Embelica officinalis*) are commonly used herbs in several herbal preparations. In Gujarat region, the leading brands in the market survey were found to be Mentat (The Himalaya Drug Company), Shankhpushpi (Zandu) and Shankhpushpi Syrup (Unjha Pharmacy), etc. Twelve Ayurvedic formulations were found to be used commonly as vitality-enhancing medicines, among which Chyavanprash (Zandu) was found to be a leading brand.

Recent research on *Bacopa monnieri* whole plant-standardized dry extract suggested that *B. monnieri* has potential for safely enhancing cognitive performance in the elderly patients.[32] Nahata *et al*. showed positive effects of *Evolvulus alsinoides* Linn. on learning behavior and memory-enhancement activity in rodents.[33]

However, these claims must be critically evaluated in terms of modern scientific parameters. These plant-derived products should be carefully standardized and their efficacy and safety for a specific application should be demonstrated. When such a project is envisaged, some prerequisites must be met. It is important to carefully correlate the disease description in the ancient literature with the modern etiology and clinical picture to ensure correct correspondence. For that, a similar pharmacoepidemiological study on doctors and patients also would be beneficial.

## APPENDIX

### Viewpoints of shopkeepers / pharmacist

Types of stores:

- General medical store
- Ayurvedic store

Location of the stores:

- Rural
- Urban

1. How many drugs are sold on the basis of
  a. Prescription Nos.__________/ day/ week
  j. Without prescription Nos.__________/ day/ week
2. What is the percentage of the sale of the herbal/ allopathic products in your store per say?
  Herbal:
  Allopathic:
3. Which companies are top five in selling of herbal/ allopathic drugs at your store?
  Herbal   Company Name   Product Name
  1.
  2.
  3.
  4.
  5.
  Allopathic   Company Name   Product Name
  1.
  2.
  3.
  4.
  5.
4. Do you give instructions to the patients?
  Yes/ No
  What instructions
5. What are group asks for these drugs?
  a. 1 to 18 years
  b. 18 to 40 years
  c. 40 to 60 years
  d. 40 to 60 years
6. What professional group asks for these drugs?
  a. School children / college students
  b. Housewives
  c. Businessmen/ service class
  d. Geriatric patients

## References

[1] Dhingra D, Parle M, Kulkurni SK (2003). Medical plants and memory. Indian Drugs.

[2] Vas CJ, Pinto C, Panikkar D, Noronha S (2001). Prevalence of dementia in an urban Indian population. Int Psychogeriatric.

[3] Larriviere D, Williams MA, Rizzo M, Bonnie RJ (2009). On behalf of the AAN Ethics, Law and Humanities Committee. Responding to requests from adult patients for neuroenhancements: Guidance of the Ethics, Law and Humanities Committee. Neurology.

[4] Farah MJ (2005). Neuroethics: The practical and the philosophical. Trends Cogn Sci.

[5] McCabe SE, Teter CJ, Boyd CJ, Knight JR, Wechsler H (2005). Nonmedical use of prescription opioids among U.S. college students: Prevalence and correlates from a national survey. Addict Behav.

[6] McCabe SE, Teter CJ, Boyd CJ, Guthrie SK (2004). Prevalence and correlates of illicit methylphenidate use among 8^th^, 10^th^, and 12^th^ grade students in the United States, 2001. J Adolesc Health.

[7] Maher B (2008). Poll results: Look who’s doping. Nature.

[8] Farah MJ, Illes J, Cook-Deegan R, Gardner H, Kandel E, King P (2004). Neurocognitive enhancement: What can we do and what should we do?. Nat Rev Neurosci.

[9] Yesavage JA, Mumenthaler MS, Taylor JL, Friedman L, O’Hara R, Sheikh J (2002). Donepezil and flight simulator performance: Effects on retention of complex skills. Neurology.

[10] Gron G, Kirstein M, Thielscher A, Riepe MW, Spitzer M (2005). Cholinergic enhancement of episodic memory in healthy young adults. Psychopharmacology.

[11] Mehta MA, Owen AM, Sahakian BJ, Mavaddat N, Pickard JD, Robbins TW (2000). Methylphenidate enhances working memory by modulating discrete frontal and parietal lobe regions in the human brain. J Neurosci.

[12] Elliott R, Sahakian BJ, Matthews K, Bannerjea A, Rimmer J, Robbins TW (1997). Effects of methylphenidate on spatial working memory and planning in healthy young adults. Psychopharmacology.

[13] Turner DC, Robbins TW, Clark L, Aron AR, Dowson J, Sahakian BJ (2003). Relative lack of cognitive effects of methylphenidate in elderly male volunteers. Psychopharmacology.

[14] Girodon F, Galan P, Monget AL, Boutron-Ruault MC, Brunet-Lecomte P, Preziosi P (1999). Impact of trace elements and vitamin supplementation on immunity and infections in institutionalized elderly patients: A randomized controlled trial. MIN. VIT. AOX. Geriatric network. Arch Intern Med.

[15] Girodon F, Lombard M, Galan P, Brunet-Lecomte P, Monget AL, Arnaud J (1997). Effect of micronutrient supplementation on infection in institutionalized elderly subjects: A controlled trial. Ann Nutr Metab.

[16] Chandra RK, Puri S (1985). Nutritional support improves antibody response to influenza virus vaccine in the elderly. Br Med J (Clin Res Ed).

[17] Chandra RK (1992). Effect of vitamin and trace-element supplementation on immune responses and infection in elderly subjects. Lancet.

[18] Jain AL (2002). Influence of vitamins and trace-elements on the incidence of respiratory infection in the elderly. Nutr Res.

[19] Graat JM, Schouten EG, Kok FJ (2002). Effect of daily vitamin E and multivitamin-mineral supplementation on acute respiratory tract infections in elderly persons: A randomized controlled trial. JAMA.

[20] Bogden JD, Bendich A, Kemp FW, Bruening KS, Shurnick JH, Denny T (1994). Daily micronutrient supplements enhance delayed-hypersensitivity skin test responses in older people. Am J Clin Nutr.

[21] Schlebusch L, Bosch BA, Polglase G, Kleinschmidt I, Pillay BJ, Cassimjee MH (2000). A double-blind, placebo-controlled, double-centre study of the effects of an oral multivitamin-mineral combination on stress. S Afr Med J.

[22] Humber JM (2002). The role of complementary and alternative medicine: Accommodating pluralism. *J Am Med Assoc*.

[23] Govindarajan R, Vijayakumar M, Pushpangadan P (2005). Antioxidant approach to disease management and the role of ‘Rasayana’ herbs of Ayurveda. J Ethnopharmacol.

[24] Brahma SK, Debnath PK (2003). Therapeutic importance of Rasayana drugs with special reference to their multi-dimensional actions. Aryavaidyan.

[25] Rege NN, Thatte UM, Dahanukar SA (1999). Adaptogenic properties of six rasayana herbs used in Ayurvedic medicine *Phytother Resnone*.

[26] Babara M, Murphy L, Frigo C (1993). Hospital Pharm.

[27] Shah JS, Anand IS (2003). Memory enhancing herbal medicines in Indian market: A study. 17^th^ IPS Annual conference (Gujarat chapter).

[28] Dev S (1997). Ethnotherapeutics and modern drug development: The potential of Ayurveda. Curr Sci.

[29] (2009). Psychotropic drugs and expanded uses: An ethical Perspective. Position Statement: Summary and recommendations adopted at the 40th meeting of the commission de l’éthique de la science et de la technologie.

[30] Izzo AA, Borrelli F, Capasso R (2002). Herbal medicine: The dangers of drug interaction. Trends Pharmacol Sci.

[31] Arria AM, Wish ED (2006). Non-medical use of prescription stimulants amongst students. Pediatr Ann.

[32] Calabrese C, Gregory WL, Leo M, Kraemer D, Bone K, Oken B (2008). Effects of a standardized *bacopa monnieri* extract on cognitive performance, anxiety, and depression in the elderly: A randomized, double-blind, placebo-controlled trial. J Altern Complement Med.

[33] Nahata A, Patil UK, Dixit VK (2010). Effects of Evolvulus alsinoides Linn. On learning behavior and memory enhancement activity in rodents. Phytother Res.

